# Social participation and healthy ageing: a neglected, significant protective factor for chronic non communicable conditions

**DOI:** 10.1186/1744-8603-7-43

**Published:** 2011-10-28

**Authors:** Wendy R Holmes, Jennifer Joseph

**Affiliations:** 1 Burnet Institute, 85 Commercial Road, Melbourne, Victoria, Australia 3004, GPO Box 2284, Melbourne, Victoria, 3001, Australia; 2 PALM Foundation, 133 Lady MacCullum's Road, Hawa Eliya, Nuwara Eliya, Sri Lanka

**Keywords:** social participation, healthy ageing, non-communicable diseases, chronic conditions

## Abstract

**Background:**

Low and middle income countries are ageing at a much faster rate than richer countries, especially in Asia. This is happening at a time of globalisation, migration, urbanisation, and smaller families. Older people make significant contributions to their families and communities, but this is often undermined by chronic disease and preventable disability. Social participation can help to protect against morbidity and mortality. We argue that social participation deserves much greater attention as a protective factor, and that older people can play a useful role in the prevention and management of chronic conditions. We present, as an example, a low-cost, sustainable strategy that has increased social participation among elders in Sri Lanka.

**Discussion:**

Current international policy initiatives to address the increasing prevalence of non-communicable chronic diseases are focused on cardiovascular disease, diabetes, respiratory disease and cancers, responsible for much premature mortality. Interventions to modify their shared risk factors of high salt and fat diets, inactivity, smoking and alcohol use are advocated. But older people also suffer chronic conditions that primarily affect quality of life, and have a wider range of risk factors. There is strong epidemiological and physiological evidence that social isolation, in particular, is as important a risk factor for chronic diseases as the 'lifestyle' risk factors, yet it is currently neglected. There are useful experiences of inexpensive and sustainable strategies to improve social participation among older people in low and lower middle income countries. Our experience with forming Elders' Clubs with retired tea estate workers in Sri Lanka suggests many benefits, including social support and participation, inter-generational contact, a collective voice, and facilitated access to health promotion activities, and to health care and social welfare services.

**Summary:**

Policies to address the increase in chronic non-communicable diseases should include consideration of healthy ageing, conditions that affect quality of life, and strategies to increase social participation. There are useful examples showing that it is feasible to catalyse the formation of Elders' Clubs or older people's associations which become self-sustaining, promote social participation, and improve health and well-being of elders and their families.

## Background

Governments and the World Health Organization have recognised the huge burden of preventable disease, disability, death and distress caused by the non communicable diseases (NCDs). Advocacy by the World Health Organization has recently pushed NCDs up the international health agenda. In September 2011 world leaders discussed the Prevention and Control of NCDs at the United Nations General Assembly in New York [1]. WHO focuses on four conditions (cardiovascular disease, diabetes, cancer, and chronic respiratory disease) responsible for most premature mortality, and four 'lifestyle' risk factors (smoking, harmful alcohol use, lack of physical activity, and high salt, high fat diets) [2]. The reasons for the epidemic of NCDs and their increasing proportion of the global burden of disease are changes in social and living conditions accompanying globalization and urbanisation, the rapid ageing of populations, and successes in reducing infectious diseases [3].

The ageing of populations, especially in Asian countries, is a result of the demographic transition caused by increases in life expectancy and declines in fertility rates. It is happening at a much faster rate in developing countries. In developed countries the proportion over 60 years of age increased from 7% to 14% over a century; many Asian countries are making the same transition in only 25 years [4]. This is also occurring at a time of other transitions, including globalisation, with migration, modern influences, urban living, smaller families with changes in traditional roles, and women working outside the home. By 2020 it is predicted that 67% of the global population over 60 years will live in developing countries [4].

Older people play important social, cultural and economic roles in their families and communities. They look after grandchildren enabling both parents to work outside the home, they undertake domestic and horticultural work, they buffer the effect of modern influences on young people, and pass on traditional rituals, skills and knowledge. They can provide emotional support, and a sense of continuity and belonging. But their contribution is often limited by chronic illness and disability. When elders become dependent the burden of care falls on family members, usually women or girls. Health care expenses for non-communicable diseases often impoverish families [5]. The rapid ageing of populations is a significant development issue [6]. However, healthy and active ageing continues to be given low priority in international public health and social policy arenas.

There is strong evidence that higher levels of social integration are associated with lower morbidity and mortality rates [7]. A recent meta-analysis to determine the extent to which social relationships influence risk for mortality found a 50% increased likelihood of survival for participants with stronger social relationships, an influence comparable with the 'lifestyle' risk factors [8].

In this paper we argue that social participation as a protective factor, deserves much greater attention, and that older people can play a useful role in the prevention and management of chronic conditions. We present the evidence that social isolation is a significant risk factor for NCDs, and discuss new understanding about the mechanisms through which isolation interacts with stress to harm the body. We argue that addressing social isolation among older people in low income settings is feasible and worthwhile. To show this we present an example from a healthy ageing project in Sri Lanka of a low-cost strategy that has increased social participation in a sustainable way, with broader benefits than anticipated. We also describe how others have successfully used similar approaches in varied settings.

## Discussion

### Social isolation a significant risk factor

Since the 1970s there has been a growing understanding of the influence of social relationships on the prevention and management of chronic conditions [9]. Higher levels of social integration have been found to provide protective effects against a wide range of physical and mental illnesses and to facilitate recovery from disease [7].

Having friends, and participating socially, can help to soften the stresses of life and reduce feelings of helplessness. The very large INTERHEART case control study across 52 countries found that the presence of psychosocial stressors was associated with increased risk of acute myocardial infarction [10]. This was still significant after adjusting for other cardiovascular risk factors. The effect of stress was independent of socioeconomic status and smoking, and occurred across all geographic regions and age groups, and in both men and women. The authors concluded that approaches aimed at modifying psycho-social stress should be developed.

In a US study, loneliness was prospectively associated with increased risk of incident coronary heart disease, after controlling for multiple confounding factors [11]. A study of Thailand rural elders found that social support buffered the impact of disability and reduced the risk of depression [12]. Studies also show that helping others helps older people to adjust to their own decline in function and health [13].

Recently Holt-Lunstad et al undertook a helpful review and appraisal of the many studies that have examined the influence of social isolation [8]. They concluded that people with adequate social relationships have a 50% greater likelihood of survival compared to those with poor or insufficient social relationships. The effect is so great that it is comparable with quitting smoking and exceeds the better publicized risk factors such as obesity and lack of physical exercise. We should also note, however, that relationships that are unhappy with conflict or excessive demands can increase risk of depression or angina [7]. Holt-Lunstad et al urge that "Social relationship-based interventions represent a major opportunity to enhance not only the quality of life but also survival."[8].

### How social isolation influences morbidity and mortality

We have evolved as a social species. In stone age times those who were isolated from others were less likely to survive - so we are primed for social contact [14]. How we behave, what we experience, and how we understand this, influences the sympathetic nervous system, and levels of hormones, such as cortisol, which in turn affect blood pressure and the immune system, making us vulnerable to a range of illnesses [15]. On the other hand, although not yet well understood, oxytocin, which appears to both prompt and be stimulated by social contact [16-18], has anti-inflammatory and cardio-protective properties [19].

Many recent studies have added to our understanding of the pathways through which social isolation influences physiological mechanisms to cause disease. Grant et al explored the effect of social isolation on the body's ability to recover from physiological responses to stress [20]. Men and women who were socially isolated had slower post-task recovery of systolic blood pressure and greater cortisol output over the day; the men also had a higher cholesterol response to stress. In a European study salivary cortisol responses to mental stress were associated with coronary artery calcification in healthy men and women [21]. In a US cross-sectional study, level of social integration was associated with fibrinogen concentration in elderly men [22]. In another US study social integration was found to modify physiologic pathways influenced by stress, such as blood pressure, reducing risk of cardiovascular disease [23].

Friendships, helping others, and social participation increase self-esteem and well-being - older people are then more likely to be motivated to change behaviours that jeopardise their health, such as smoking and drinking, and to maintain their healthier behaviours. They are more likely to seek health care, and to have better self-care in the management of their conditions.

### Prevention of chronic non-communicable diseases

The 'Package of Essential Non-communicable Disease Interventions for Primary Health Care in Low-Resource Settings' was developed by WHO to improve access to cost effective interventions in resource constrained settings. It addresses the risk factors of smoking, diets high in fat and salt, lack of physical activity, and high alcohol intake. The 'package' does not include strategies to encourage social participation.

Simple messages are effective for advocacy purposes. So the choice by WHO of the 4 × 4 concept (4 diseases; 4 shared risk factors) is understandable [24]. However, this sharp focus on the 'lifestyle' risks tends to keep other significant factors, such as social participation, in the shadows. The focus on 'lifestyle' factors can also suggest that individuals are responsible for their own behaviours and the illnesses that result from them. Yet the evidence that low birth weight and epigenetic factors influence vulnerability to diabetes and cardiovascular disease is very relevant in low income settings [25].

There are a number of possible reasons why the potential of encouraging social participation has not been recognised and promoted within the WHO package for addressing NCDs. Understandably WHO has a commitment to evidence-based policy making, which tends to prioritise results of randomised controlled trials. Much of the evidence for the influence of social participation on development of non-communicable diseases, and understanding of the mechanisms for this association, are relatively recent, so there have been few trials of interventions. It is difficult to standardise such interventions for trials, and they are likely to be context-dependent. However, because an intervention has not been trialled does not mean that it may not be effective - there is a difference between 'no evidence' and 'evidence of no effect'. It has been pointed out that rigorous systematic review may eliminate much relevant information [26]. Considering only interventions proven to be cost-effective for a particular outcome also fails to take into account that strategies such as promoting social participation have a strong evidence-based rationale and have multiple benefits.

### Promotion of healthy ageing

Although rapid population ageing is recognized as one of the reasons that NCDs are increasing, there have been few attempts to integrate the responses needed for prevention and management of NCDs and those needed to promote healthy ageing. At the WHO web-site, for example, the page for the Department of Chronic Disease and Health Promotion [27] has no links to the healthy ageing resources and the Department of Ageing and Life Course page has no links to the topic of non-communicable or chronic diseases [28]. This arrangement tends to be mirrored in developing countries where within a Ministry of Health there is often one department addressing NCDs and another responsible for healthy ageing, resulting in duplication and gaps in policy and guidelines. Efforts to prevent the four highlighted NCDs throughout the life course will certainly prevent much illness and disability among older people. Nevertheless, with the focus on the burden of premature mortality (that is, deaths before 60 years) there is a risk that common chronic conditions that affect the quality of life of older people, their families and caregivers will not receive the attention they deserve. The current cohort of older women in low and middle-income countries have tended to experience high parity and poor access to obstetric care increasing the risk of stress incontinence and prolapse [29]. A long life of carrying heavy loads leads to low back pain causing severe functional disability [30]. In old age immunity is reduced increasing risk for infectious diseases [31]. Poor oral and dental health, poverty, a desire to give their food to their children or grandchildren, and lack of access to a variety of foods all adversely affect nutritional status [31,32]. Long exposure to indoor smoke and occupational hazards can result in chronic respiratory diseases. Depression and dementia are common, but may not be thought of as health conditions requiring treatment or care, and counselling and support are rare [33]. Widowhood contributes to poverty and depression.

The "fair innings" argument has sometimes been used to suggest that because older people have 'had their turn' available resources should be invested in productive adults and the potential of children. Older people's considerable economic contribution, through the child care, domestic and agricultural work they perform, does not appear in national accounts. The Disability Adjusted Life Years measure used to quantify the burden of disease has built in "value choices" weighing a year of healthy life lived at very young ages and older ages lower than years lived in between [34]. Despite older people being the poorest and most vulnerable population group, healthy ageing was not included among the Millennium Development Goals.

### Case study - addressing social participation of elders in tea estate communities in Sri Lanka

Sri Lanka is a lower middle income country with one of the fastest growing populations of older people in the world due to early gains in life expectancy and reduction in fertility rates [35]. Currently those over 60 years make up about 12% of the population, and this is expected to increase to over 20% of the population by 2030 [35]. The Sri Lankan Ministry of Social Services, the National Council for Elders, and the Ministry of Health have been responding to this issue, and through a consultative process have developed a National Action Plan on Ageing [36]. A project that aims to improve the health and well-being of elders in the tea estate sector has provided useful lessons about how to increase social participation. The project, funded by AusAID, is a collaboration between the PALM Foundation, a local community development non-government organisation in Nuwara Eliya, and Burnet Institute, an international health research institute in Australia. The project, which began in 2004, covers a population of about 50,000 predominantly Tamil tea estate workers and adjacent Sinhala villagers with 4,000 elders over 60 years and their family members, in the district of Nuwara Eliya. Our baseline survey showed that 57% are women, and most are young old, that is 45% were between 60 and 65, and 28% between 66 and 70 years; only about 4% were over 80. In the 19^th ^century the British brought workers from India to work in the tea estates of Ceylon; they have remained a socio-economically disadvantaged group.

The retired tea estate workers had little or no income, poor and crowded living conditions, and limited access to services. Many were reported to be less socially engaged than before their retirement. PALM Foundation and the Burnet Institute have a participatory approach, so one of the project strategies was to establish Elders' Clubs. PALM community mobilisers first made a register of older people in their estate communities and consulted them about forming Elders' Clubs. At the first meetings the elders mapped households where elders lived, including those bedridden or disabled. The Elders' Clubs chose two leaders, a woman and a man, and a name for their Club. They arrange monthly meetings and a variety of activities. There are currently 55 Clubs with a total of 3,913 members. Participatory project evaluations, with focus group discussions with elders and interviews with officials, community mobilisers and the project team, have found that the strategy was successful at promoting social participation and has had wider benefits than anticipated.

*Greater social contact: *Activities such as playing music, dance competitions, sports, oral history and excursions to religious sites have provided greater social contact between elders. This has led to increased self-esteem, more friendships, and better relationships within families.

*"Now we live in unity. Earlier when we go on the road we don't recognise other elders, but now we are like brothers and sisters" *[Male elder].

Opportunities to practice religious rituals together are often especially important to older people. Ritual provides meaning, a sense of familiarity, belonging and continuity, opportunity to meet regularly with others, and motivation. Elders now organize their own activities:

*"A cultural competition was organized - many took part for the first time. Some of the women were saying that they danced forgetting themselves. It was one of the happiest days of their lives." *[Field staff member]

*Greater social support: *Club members visit sick or bereaved peers, often giving pooled donations, and have organised their own saving and small loan schemes.

*"When I was sick last month, five members of our club visited me at home. I felt very happy and safe during that time. And also they offered a pooja (prayer) at our kovil (temple) on behalf of me. I think that's why I recovered soon*". [Male elder]

*Improved access to services: *Through Elders' Club meetings illiterate members were assisted to obtain identity cards which enable them to access welfare entitlements. Club meetings also facilitated the organization of eye and oral health screening (with help from HelpAge Sri Lanka) with referral for cataract surgery or dental treatment. The screening data has enabled advocacy with government services, for example, to treat the backlog of cataract blindness. Treating preventable blindness has great impact on quality of life of both elders and their family members:

*"In Mahauva in a family one person was paid to look after the elder who had cataract. Now after surgery there is no need for a person to look after him." *[Community mobiliser]

*"We have come from darkness to light" *[Male elder]

*"When I was blind I felt like my hands and legs are not functional, now (after surgery) I can walk well and go anywhere, that is why I could come for this discussion too" *[Female elder]

*Greater community participation: *Leadership skills training and inter-generational activities with young people have resulted in greater community participation and respect for elders.

*"Many opportunities have reached the elders who are involved in PALM project; it has changed them psychologically; they have come out from their houses" *[Grama Niladari (Local administrative officer)]

*"Our way of dressing, behaviour etc, have totally changed from how it was in at the beginning, we feel like studying at schools. We are not elders, we feel like students" *[Male elder]

*"We are amazed at ourselves. We feel like youth" *[Male elder]

More youth and children are helping elders, for example, in repairing latrines, helping in watering vegetable gardens, accompanying elders to the hospital for cataract surgery, and helping with preparing and serving tea at elders' meetings.

*"They tell stories, they sing lullabies, they take us to temple festivals, they advise us not be involved in bad habits - if only they live better they will look after us" *[Young person]

*Opportunities for health promotion: *Club meetings provide opportunities for interactive health promotion sessions and have allowed the identification and training of peer educators who provide information and support about chronic conditions such as diabetes and hypertension to all age groups; those who have had cataract surgery have encouraged others to attend for surgery who were previously reluctant. This has improved knowledge and care seeking behaviour:

*"The older people said that their knowledge and understanding of diseases has improved. They tend to seek medical advice more than before. Their beliefs have changed and they have realized the importance of managing some of the non communicable diseases." *[PALM team member]

*Increased leadership: *The Clubs have grown in strength and independence and the elders soon took over managing their own clubs.

*"I am very proud of being of a leader, I have been able to get walking sticks to four people, six people have undergone surgery and they are seeing well. 16 persons have received spectacles, one person got a wheel chair. All of us have gone on trips, I am the one who organized all these and this gives me satisfaction*". [Club Leader]

However, taking on leadership is not without its challenges and conflicts, particularly in relation to saving and small loan schemes. The leaders have their own monthly regional meeting to support and learn from each other. Clubs are vulnerable to illness, death and migration of leaders and members. Some members have domestic commitments or jobs that limit their involvement. Club leaders helped to develop their own evaluation criteria to identify weak clubs, which are invited to visit and learn from stronger clubs, with good results. The clubs have made their own savings and opened bank accounts, allowing them to apply successfully for government registration, which then entitles them to certain benefits. The registration of the clubs has increased their status and their meetings and events are now often attended by the Grama Niladari (local administrative officer), and estate management. There has been steady progress towards sustainability, with many stories demonstrating increasing strength and independence.

*"The elders of Mayfield estate decided that they would publicize their active Elders' Club. They organized a sports event and decided to present gifts to poor elders. But funds were a problem. They approached business people and others and collected around Rs 10,000 (~US$100) in cash and kind. Representatives of the central provincial council, other government officers, officers of the estate management, school heads etc. were invited. They did it in a big way. It was amazing that the Elders' Club themselves, with the CBO, organized this kind of a program. Most of all the people from outside came to know of this Elders' Club." *[PALM project coordinator]

*Greater visibility and a collective voice: *Elders now have greater visibility, for example, estate management and the estate community based organizations are recognising and responding to their needs:

*"In Dayagama West 5^th ^a water project was implemented and usually people get one tap for five houses. To get an individual connection it will cost Rs.2,500.00. There were two elders who could not pay for this. The CBO considered their situation, gave them connection to their house, and it bore the cost." *[Field team member]

*"13 elders had to climb a difficult and slippery path to get to toilets. With the involvement of the CBO a flight of stairs was constructed with railings to hold and now the elders safely go to the toilet*." [Field team member]

Through their clubs the elders now have a collective voice to influence politicians, government services and estate management. Some Clubs have made their own official letterheads to write about their needs.

*"Medawatha elders have written a letter to the local council member requesting a common gathering hall for them, and Maha-Ouvah elders have written a letter to a Provincial Council member who was selected in the last elections, informing their activities and requests." *[Field team member]

The success of these efforts to encourage greater social participation by elders was assisted by the familiarity of PALM Foundation workers with their own communities. They understand the social and political dynamics and have been able to suggest new ways ahead when problems are identified. The partnership with Burnet Institute has enabled contribution of new ideas from outside, for example, the use of picture cards for health promotion, and the project coordinator attended a two week program on healthy ageing in Melbourne where she was able to get to know senior Sri Lankan health officials and academics, as well as learn about relevant research findings in other countries in the region. The partnership has also increased research skills capacity and aided in dissemination of the lessons learned from the project.

This year in Sri Lanka the Protection of the Rights of Elders Act of 2000 was amended to include: "establish an Elders Committee in every Grama Niladhari Division, Divisional Secretariat's Divisions, Administrative District and Provincial Council area." The process of establishing self-sustaining Elders' Clubs described in this case study provides useful lessons for the National Secretariat for Elders in implementing this goal, and for other low income settings. It has also shown the valuable role local non-government organisations can play in linking with different government sectors to provide more appropriate services and in assisting older people to access services.

The international non-government organisation, HelpAge International, has also had successful experiences of establishing Older People's Associations in varied settings across South and South East Asia, and has similarly found a range of beneficial health and social outcomes [37]. With increasing urbanization it is also important to consider strategies for social participation in urban settings [38]. A Red Cross Society project in Kutaisi, Georgia, found that socially isolated and poor older people they consulted wanted somewhere to meet. Once the older people started to attend a club their self-esteem increased, they made friends, helped each other, took better care of their own health, and became a pressure group able to influence local officials and politicians [39]. Older people often have more time than younger adults to participate in social activities, and often have the interest, skills and wisdom to contribute to the organisation and management of their clubs.

## Summary

Many low and lower middle income countries, especially those in South and South-East Asia, have rapidly ageing populations. National governments are seeking guidance in promoting healthy and active ageing. There is much evidence that social isolation, lack of support and stress increase risk of morbidity and mortality from chronic conditions. Yet strategies to increase opportunities for social participation have not been emphasised in current international policy responses to the increase in chronic conditions. That the agendas of addressing NCDs and promoting healthy ageing overlap has not been sufficiently recognised, and attending to the health problems of older people has been relatively neglected. There are several useful examples of successful and sustainable initiatives of catalysing the formation of elders' clubs or older people's associations. Benefits include greater social contact, social support, opportunities for learning, increased and easier access to health and social welfare services, better self-management of chronic conditions, greater participation in the community with inter-generational benefits, better relationships within families, greater visibility and increased influence. Support should be provided for further research to assess the feasibility and impact of social participation strategies on prevention and management of chronic conditions. Policy advice should promote encouragement of links between government services and local non-government organisations that are well placed to facilitate social organisations of older people. When in good health older people can be of great benefit to their families and communities.

## Competing interests

The authors declare that they have no competing interests.

## Authors' contributions

WH reviewed the literature and drafted the paper; Both authors were responsible for planning and implementing the project on which the case study is based; JJ coordinated the project and evaluations, and provided ideas for the paper. Both authors read and approved the final manuscript.

## Authors' information

Dr Wendy Holmes is Principal Fellow - Healthy Ageing at the Burnet Institute for Medical Research and Public Health, Melbourne, Australia.

Ms Jennifer Joseph is Deputy Team Leader and Coordinator of the Healthy Ageing Project at PALM Foundation, Nuwara Eliya.

## References

[B1] UN General Assembly Political Declaration of the High-level Meeting of the General Assembly on the Prevention and Control of Non-communicable Diseases 2011 UN New York

[B2] WHO 2008-2013 Action plan for the global strategy for the prevention and control of non-communicable diseases 2009 Geneva

[B3] Stuckler D Population causes and consequences of leading chronic diseases: a comparative analysis of prevailing explanations Milbank Q 2008 86 2 273 326 10.1111/j.1468-0009.2008.00522.x 18522614 PMC2690359

[B4] Shrestha LB Population ageing in developing countries Health Affairs 2000 19 3 204 212 10.1377/hlthaff.19.3.204 10812800

[B5] WHO Global status report on non-communicable diseases 2010 2011 Geneva

[B6] HelpAge International Ageing: the forgotten development issue Ageing and development 1998 1 http://www.helpage.org/download/4d067106d9d75

[B7] Seeman T Health promoting effects of friends and family on health outcomes in older adults Am J Health Promot 2000 14 362 370 11067571 10.4278/0890-1171-14.6.362

[B8] Holt-Lunstad J Smith TB Layton JB Social relationships and mortality risk: A meta-analytic review PLoS Med 2010 7 e1000316 10.1371/journal.pmed.1000316 20668659 PMC2910600

[B9] House JS Landis KR Umberson D Social relationships and health Science 1988 241 540 545 10.1126/science.3399889 3399889

[B10] Rosengren A Hawken S Ounpuu S Sliwa K Zubaid M Almahmeed WA Blackett KN Sitthi-amorn C Sato H Yusuf S INTERHEART investigators Association of psychosocial risk factors with risk of acute myocardial infarction in 11119 cases and 13648 controls from 52 countries (the INTERHEART study): case-control study Lancet 2004 364 953 62 10.1016/S0140-6736(04)17019-0 15364186

[B11] Thurston RC Kubzansky LD Women, loneliness, and incident coronary heart disease Psychosom Med 2009 71 8 836 42 10.1097/PSY.0b013e3181b40efc 19661189 PMC2851545

[B12] Suttajit S Punpuing S Jirapramukpitak T Tangchonlatip K Darawuttimaprakorn N Stewart R Dewey ME Prince M Abas MA Impairment, disability, social support and depression among older parents in rural Thailand Psychol Med 2010 40 1711 21 10.1017/S003329170999208X 20056022 PMC2928999

[B13] Greenfield EA Felt obligation to help others as a protective factor against losses in psychological well-being following functional decline in middle and later life J Gerontol B Psychol Sci Soc Sci 2009 64 723 32 19825942 10.1093/geronb/gbp074 PMC3270305

[B14] Cacioppo JT Hawkley LC Norman GJ Berntson GG Social isolation Ann NY Acad Sci 2011 1231 17 22 21651565 10.1111/j.1749-6632.2011.06028.x PMC3166409

[B15] McEwen BS Gianaros PJ Central role of the brain in stress and adaptation: Links to socioeconomic status, health, and disease Ann NY Acad Sci 2010 1186 1 190 222 10.1111/j.1749-6632.2009.05331.x 20201874 PMC2864527

[B16] Seltzer LJ Ziegler TE Pollak SD Social vocalizations can release oxytocin in humans Proc Biol Sci 2010 277 2661 6 10.1098/rspb.2010.0567 20462908 PMC2982050

[B17] Gordon I Martin C Feldman R Leckman JF Oxytocin and Social Motivation Dev Cogn Neurosci 2011 1 471 493 10.1016/j.dcn.2011.07.007 21984889 PMC3185363

[B18] Taylor SE Gonzaga GC Klein LC Hu P Greendale GA Seeman TE Relation of Oxytocin to Psychological Stress Responses and Hypothalamic-Pituitary-Adrenocortical Axis Activity in Older Women Psychosom Med 2006 68 238 245 10.1097/01.psy.0000203242.95990.74 16554389

[B19] Gutkowska J Jankowski M Oxytocin Re-visited: Its Role in Cardiovascular Regulation J Neuroendocrinol 2011 in press 10.1111/j.1365-2826.2011.02235.x 21981277

[B20] Grant N Hamer M Steptoe A Social isolation and stress-related cardiovascular, lipid, and cortisol responses Ann Behav Med 2009 37 1 29 37 10.1007/s12160-009-9081-z 19194770

[B21] Hamer M O'Donnell K Lahiri A Steptoe A Salivary cortisol responses to mental stress are associated with coronary artery calcification in healthy men and women Eur Heart J 2010 31 424 9 10.1093/eurheartj/ehp386 19744954

[B22] Loucks EB Berkman LF Gruenewald TL Seeman TE Social integration is associated with fibrinogen concentration in elderly men Psychosom Med 2005 67 3 353 8 10.1097/01.psy.0000160482.89163.e8 15911896

[B23] Troxel WM Buysse DJ Hall M Kamarck TW Strollo PJ Owens JF Reis SE Matthews KA Social integration, social contacts, and blood pressure dipping in African-Americans and whites J Hypertens 2010 28 2 265 71 10.1097/HJH.0b013e328333ab01 20051909 PMC2864490

[B24] WHO 2008-2013 Action plan for the global strategy for the prevention and control of noncommunicable diseases 2009 Geneva

[B25] Tarry-Adkins JL Ozanne SE Mechanisms of early life programming: current knowledge and future directions Am J Clin Nutr 2011 in press 10.3945/ajcn.110.000620 21543536

[B26] Kemm J The limitations of 'evidence-based' public health J Eval Clin Pract 2006 12 319 24 10.1111/j.1365-2753.2006.00600.x 16722916

[B27] WHO Department of Chronic Diseases and Health Promotion http://www.who.int/chp/en/

[B28] WHO Healthy Ageing through the Life Course http://www.who.int/topics/ageing/en/

[B29] Senanayake P Women and reproductive health in a graying world Int J Gynecol Obstet 2000 70 59 67 10.1016/S0020-7292(00)00224-1 10884534

[B30] Hoy D March L Brooks P Woolf A Blyth F Vos T Buchbinder R Measuring the global burden of low back pain Best Pract Res Clin Rheumatol 2010 24 155 65 10.1016/j.berh.2009.11.002 20227638

[B31] Meydani A Ahmed T Meydani SN Aging, nutritional status, and infection in the developing world Nutrition Reviews 2005 63 7 Washington 233 247 10.1111/j.1753-4887.2005.tb00379.x 16121477

[B32] Petersen PE Yamamoto T Improving the oral health of older people: the approach of the WHO Global Oral Health Programme Community Dent Oral Epidemiol 2005 33 81 92 10.1111/j.1600-0528.2004.00219.x 15725170

[B33] Patel V Prince M Ageing and mental health in a developing country: who cares? Qualitative studies from Goa, India Psychol Med 2001 31 1 29 3 10.1017/S0033291799003098 11200957

[B34] Anand S Hanson K Disability-adjusted life years: a critical review J Health Econ 1997 16 685 702 10.1016/S0167-6296(97)00005-2 10176779

[B35] World Bank Human Development Unit, South Asia Region Report No. 43396-LK Sri Lanka addressing the needs of an aging population May 28, 2008 http://siteresources.worldbank.org/INTSRILANKA/.../LKAgingFullRep.pdf

[B36] Ministry of Social Services; National Council for Elders National Action Plan on Ageing 2011-2015 2011 Colombo, Sri Lanka

[B37] HelpAge International Older people in community development: The role of older people's associations in enhancing local development 2009 HelpAge International East Asia/Pacific Regional Development Centre, Chiang Mai http://www.helpage.org/resources/publications/

[B38] Plouffe L Kalache A Towards global age-friendly cities: determining urban features that promote active aging J Urban Health 2010 87 5 733 9 10.1007/s11524-010-9466-0 20549569 PMC2937125

[B39] Red Cross Society Social care in Georgia http://www.redcross.org.uk/What-we-do/Health-and-social-care/Social-support-overseas/Social-care-in-Georgia

